# A Influência da Obesidade e da Atividade Física no Risco Cardiovascular

**DOI:** 10.36660/abc.20220381

**Published:** 2022-07-29

**Authors:** Claudio Leinig Pereira da Cunha

**Affiliations:** 1 Universidade Federal do Paraná Clínica Médica Curitiba PR Brasil Universidade Federal do Paraná - Clínica Médica, Curitiba, PR – Brasil

**Keywords:** Doenças Cardiovasculares, Obesidade, Fatores de Risco, Sedentarismo, Doença Arterial Periférica, Diabetes, Dislipidemias, Atividade Física, Exercício, Estilo de Vida

A doença cardiovascular aterosclerótica (DCVA) é comum na população geral, afetando a maioria dos adultos após os 60 anos. A doença inclui quatro áreas principais: (1) Cardiopatia coronária, (2) Doença cérebro vascular, (3) Doença arterial periférica e (4) Aterosclerose aórtica com aneurismas.^1^ As condições que tradicionalmente são associadas à instalação de DCVA (os chamados “fatores de risco”) são as dislipidemias, diabetes mellitus, hipertensão arterial, tabagismo, obesidade, sedentarismo e histórico familiar de DCVA.^2^ As alterações vasculares ateroscleróticas podem começar na infância, preparando o cenário para eventos cardiovasculares na vida adulta.^3^ Tornquist et al.,^4^ apresentam neste número dos Arquivos Brasileiros de Cardiologia alguns aspectos da obesidade e da aptidão cardiorrespiratória em relação ao risco cardiometabólico em crianças.

A obesidade é um problema de saúde pública que tem se expandido no mundo inteiro. De acordo com relatório da Organização Mundial da Saúde em 2016, a obesidade triplicou desde 1980.^5^ A prevalência de obesidade e sobrepeso aumentou também entre os jovens, passando de 16% em 1980 para 23% em 2013.^5^

De longa data a obesidade é relacionada com um risco aumentado de DCVA. Há várias alterações fisiológicas e metabólicas associadas com a obesidade que podem contribuir para o aumento deste risco: (1) Resistência à insulina e hiperinsulinemia; (2) Anormalidades no metabolismo lipídico; (3) Hipertensão arterial; (4) Remodelamento do ventrículo esquerdo; (5) Transtornos do sono; (6) Inflamação sistêmica aumentada; (7) Ativação do sistema nervoso simpático, e, (8) Disfunção endotelial.^6^

A obesidade tem sido associada com a mortalidade total em diversos estudos, assim como com a Cardiopatia Coronária, Insuficiência Cardíaca, Fibrilação Atrial e Morte Súbita.^6^

Estudos de autópsias de crianças demonstram que a obesidade se correlaciona positivamente com alterações ateroscleróticas na aorta e nas artérias coronárias durante a infância.^7^ Também, um grande estudo prospectivo dinamarquês, com 276.835 crianças nascidas entre 1930 e 1976, avaliou o Índice de Massa Corporal das crianças e observou uma relação linear positiva com o número de eventos coronarianos isquêmicos na vida adulta.^8^

Desta forma, muitas são as evidências que associam a obesidade à DCVA, desde a infância. Por outro lado, a redução do peso melhora muito os fatores de risco relacionados à obesidade: diminui a pressão arterial, reduz a incidência de diabetes, melhora o perfil lipídico, diminui a resistência à insulina, melhora a função endotelial e reduz a concentração da proteína C-reativa.^9^

O estilo de vida sedentário tem sido reconhecido como um fator de risco independente para DCVA. O incremento da atividade física se relaciona com ganho de saúde, melhor qualidade de vida e maior expectativa de vida.^2^ A atividade física envolve modalidades ocupacionais, domésticas e de lazer.^10^

Melhora da capacidade física e da qualidade de vida seriam razões suficientes para a adesão aos exercícios físicos, mas vários outros efeitos benéficos são relacionados à prática física. Contribui no controle do peso, melhora o perfil lipídico, reduz a pressão arterial, ajuda no tratamento e prevenção da diabetes mellitus, reduz a inflamação (expressa pela proteína C-reativa). O exercício influencia também o estilo de vida, diminuindo a possibilidade de fumar, reduzindo o estresse e o apetite.^11^

Os benefícios do exercício rotineiro são extremamente valiosos. Se repetem nas diversas faixas etárias, desde jovens até idosos,^12^ e são ratificados para crianças e jovens na pesquisa de Tornquist.^4^
