# Supplementary material for: A Influência da Obesidade e da Atividade Física no Risco Cardiovascular
Source: Arq Bras Cardiol. 2022 Jul 29;119(2):244–5. [Article in Portuguese] doi: 10.36660/abc.20220381 (PMC9363052; doi:10.36660/abc.20220381)
Supplement: Translation

# Translation: The Influence of Obesity and Physical Activity on Cardiovascular Risk

**Authors:** Claudio Leinig Pereira da Cunha

**Affiliations:** 1 Curitiba PR Brazil Universidade Federal do Paraná - Clínica Médica, Curitiba, PR – Brazil

**Keywords:** Cardiovascular Diseases, Obesity, Risk Factors, Sedentarism, Peripheral Artery Disease, Diabetes, Dyslipidemias, Life Style

Atherosclerotic cardiovascular disease (ACVD) is common in the general population, affecting most adults over 60 years of age. The disease includes four main areas: (1) Coronary heart disease, (2) Cerebrovascular disease, (3) Peripheral arterial disease, and (4) Aortic atherosclerosis with aneurysms.^1^ The conditions traditionally associated with the installation of ACVD (the so-called “risk factors”) are dyslipidemia, diabetes mellitus, arterial hypertension, smoking, obesity, sedentary lifestyle and a family history of ACVD.^2^ Atherosclerotic vascular changes may begin in childhood, setting the stage for cardiovascular events in adulthood.^3^ Tornquist et al.^4^ present in this issue of Arquivos Brasileiros de Cardiologia some aspects of obesity and cardiorespiratory fitness in relation to cardiometabolic risk in children.

Obesity is a public health problem that has expanded worldwide. According to a report by the World Health Organization in 2016, obesity has tripled since 1980.^5^ The prevalence of obesity and overweight also increased among young people, from 16% in 1980 to 23% in 2013.^5^

Obesity has long been associated with an increased risk of ACVD. There are several physiological and metabolic changes associated with obesity that may contribute to increased risk: (1) Insulin resistance and hyperinsulinemia; (2) Abnormalities in lipid metabolism; (3) Arterial hypertension; (4) Left ventricular remodeling; (5) Sleep disorders; (6) Increased systemic inflammation; (7) Activation of the sympathetic nervous system, and, (8) Endothelial dysfunction.^6^

Obesity has been associated with total mortality in several studies, as well as with Coronary Heart Disease, Heart Failure, Atrial Fibrillation and Sudden Death.^6^

Autopsy studies of children show that obesity is positively correlated with atherosclerotic changes in the aorta and coronary arteries during childhood.^7^ Also, a large prospective Danish study, with 276,835 children born between 1930 and 1976, evaluated the Body Mass Index of children and observed a positive linear relationship with the number of ischemic coronary events in adulthood.^8^

Thus, there is much evidence that associates obesity with ACVD since childhood. On the other hand, weight reduction greatly improves obesity-related risk factors: it lowers blood pressure, reduces the incidence of diabetes, improves the lipid profile, decreases insulin resistance, improves endothelial function, and reduces protein C-reactive concentration.^9^

A sedentary lifestyle has been recognized as an independent risk factor for ACVD. The increase in physical activity is related to health gains, a better quality of life and longer life expectancy.^2^ Physical activity involves occupational, domestic and leisure activities.^10^

Improvement in physical capacity and quality of life would be sufficient reasons for adhering to physical exercises, but several other beneficial effects are related to physical practice. It contributes to weight control, improves lipid profile, lowers blood pressure, helps treat and prevent diabetes mellitus, and reduces inflammation (expressed by C-reactive protein). Exercise also influences lifestyle, reducing the possibility of smoking, reducing stress and appetite.^11^

The benefits of routine exercise are extremely valuable. They are repeated in different age groups, from young people to the elderly,^12^ and are confirmed for children and young people in the study by Tornquist.^4^
